# miR-125b reverses cisplatin resistance by regulating autophagy via targeting RORA/BNIP3L axis in lung adenocarcinoma

**DOI:** 10.32604/or.2023.044491

**Published:** 2024-03-20

**Authors:** LEI LIU, NA GUO, XIANGLING LI, QIAN XU, RUILONG HE, LIMIN CHENG, CHUNYAN DANG, XINYU BAI, YIYING BAI, XIN WANG, QIANHUI CHEN, LI ZHANG

**Affiliations:** 1 Department of Immunology, Chengde Medical University, Chengde, 067000, China; 2 Department of Pathology, Chengde Medical University, Chengde, 067000, China; 3 Department of Oncology, The Affiliated Hospital of Chengde Medical University, Chengde, 067000, China; 4 Department of Hepatobiliary Surgery, The Affiliated Hospital of Chengde Medical University, Chengde, 067000, China

**Keywords:** Lung adenocarcinoma, miRNAs, Cisplatin, Resistance, Autophagy

## Abstract

The platinum-based chemotherapy is one of the most frequently used treatment protocols for lung adenocarcinoma (LUAD), and chemoresistance, however, usually results in treatment failure and limits its application in the clinic. It has been shown that microRNAs (miRNAs) play a significant role in tumor chemoresistance. In this study, miR-125b was identified as a specific cisplatin (DDP)-resistant gene in LUAD, as indicated by the bioinformatics analysis and the real-time quantitative PCR assay. The decreased serum level of miR-125b in LUAD patients was correlated with the poor treatment response rate and short survival time. MiR-125b decreased the A549/DDP proliferation, and the multiple drug resistance- and autophagy-related protein expression levels, which were all reversed by the inhibition of miR-125b. In addition, xenografts of human tumors in nude mice were suppressed by miR-125b, demonstrating that through autophagy regulation, miR-125b could reverse the DDP resistance in LUAD cells, both *in vitro* and *in vivo*. Further mechanistic studies indicated that miR-125b directly repressed the expression levels of RORA and its downstream BNIP3L, which in turn inhibited autophagy and reversed chemoresistance. Based on these findings, miR-125b in combination with DDP might be an effective treatment option to overcome DDP resistance in LUAD.

## Introduction

There is still a high rate of lung cancer-related death and morbidity around the world [1], and about 40% of cases are lung adenocarcinomas (LUAD) [2]. Most lung cancer patients (57%) are diagnosed in the advanced stages, for whom platinum-based chemotherapy regimens have been widely accepted as an effective treatment option in clinics, consisting of third-generation chemotherapy and platinum [3]. Chemoresistance, however, limits the clinical efficacy of these chemo agents and may thus result in poor prognosis. Therefore, it is of interest to understand the mechanism of cisplatin (DDP) resistance in LUAD treatment.

Recent evidence has proven that autophagy, a dynamic catabolic process that degrades and recycles intracellular organelles and proteins, is essential to chemoresistance. In general and normal conditions, a low level of autophagy occurs, and this process is triggered by stress stimuli, amino acid limitation, and agents present in the body, leading to reduced autolysosomal activity while increased autophagosome synthesis [4]. Autophagy has been widely accepted as a double-edged sword, which would both kill and protect cells in the progression of tumors [5,6]. In cancer chemotherapy, when the protection mechanism of autophagy is initiated, chemoresistance often occurs. Several studies have indicated that DDP activates autophagy and prevents DDP-induced apoptosis, leading to DDP resistance [7,8]. These findings strongly suggest that regulating autophagy activity can reverse chemoresistance.

RNA molecules with a length of 18–25 nt have been called microRNAs (miRNAs), which are important regulators of tumor emergence and development, thus representing the targets for chemoresistance prevention [9]. Although miRNAs play a critical role in chemoresistance by regulating various drug-resistant mechanisms, including drug transport, epithelial-mesenchymal transition (EMT), redox homeostasis, DNA damage repair, and autophagy, the exact underlying mechanisms are far from clear. Thus, finding novel miRNAs as targets for chemoresistance and clarifying the mechanisms of drug resistance is of great significance.

In this study, miR-125b was identified to be considerably downregulated in DDP-resistant LUAD cells and tissues based on the bioinformatics analysis of the Gene Expression Omnibus (GEO) chips. Importantly, miR-125b overexpression reversed the DDP resistance in non-small cell lung cancer (NSCLC) cells and inhibited the autophagy by targeting retinoic acid receptor-related orphan receptor A (RORA) and downstream autophagy-related genes BCL2 Interacting Protein 3 Like (BNIP3L). These findings highlight the regulatory significance of miR125b on autophagy and chemoresistance, thus extending the understanding of their roles, in LUAD.

## Materials and Methods

### Data acquisition and differential expression analysis

To search for DDP-resistant miRNAs in LUAD, the GEO (http://www.ncbi.nlm.nih.gov/geo/) datasets were retrieved, using the following keywords: lung adenocarcinoma cisplatin-resistant miRNA (study keyword), Series (entry type), and homo sapiens (organism). Two tissue-based datasets (GSE56036 [10] and GSE168707 [11]), and two cell-based datasets (GSE43249 and GSE157692 [12]) in DDP-sensitive and -resistant tissues/cells, were downloaded from the GEO database. The four dataset details were summarized in Suppl. Table S1. The differentially expressed genes (DEGs) were screened out using the limma package of the R software, which was visualized via the heatmap using an online tool (xiantao, https://www.xiantao.love/). Significantly differentially expressed miRNAs were screened by |log_2_(fold-change)|>1 and *p* < 0.05.

### Cell lines

The LUAD cell line A549 and DDP-resistant cell line A549/DDP were purchased from Shanghai Zhewen Biotechnology Co., Ltd. (Shanghai, China). The human bronchial epithelial cell 16HBE and human embryonic kidney 293T (HEK-293T) cells were obtained from Procell (Wuhan, Hubei, China). These cells were cultured with the RPMI 1640 medium (Gibco, Grand Island, USA), supplemented with 10% fetal bovine serum (Procell, Wuhan, China), in a 37°C, 5% CO_2_ incubator.

### Study patients and sample collection

Totally 97 LUAD patients were included herein, who received four cycles of platinum-containing chemotherapy regimen and had measurable diseases according to the Response Evaluation Criteria in Solid Tumors (RECIST, version 1.1). The patients with complete response (CR) and partial response (PR) were categorized into the chemotherapy response-sensitive group (0 CR and 55 PR), while those with stable disease (SD) and progressive disease (PD) were categorized into the chemotherapy response-resistant group (32 SD and 10 PD). Venous blood samples were collected from the included study patients, which were immediately frozen at −80°C. The study was approved by the Ethics Committee of Chengde Medical University, and the written informed consent was signed by each patient.

### Real-time quantitative PCR (RT-qPCR)

According to the manufacturer’s instructions, total RNA was extracted from samples using TRIzol (Invitrogen, Carlsbad, USA), and the cDNA was generated. PCR amplicons were carried out with the SYBR Green PCR kit (Takara, Dalian, China). The miR-125b level was detected with the Hairpin-itTM miRNA qPCR kit (genePharma, Shanghai, China). Primer sequences were listed in Suppl. Table S2. For each sample, RT-qPCR was performed on the ABI-7500 PCR system (ABI, CA, USA) in triplicate. The PCR conditions were as follows: 94°C for 3 min; 40 cycles of 94°C for 12 s, 62°C for 30 s; and 72°C for 30 s. The relative expression levels were calculated with the 2^−ΔΔCq^ method. GAPDH and U6 were used as internal references.

### Survival analysis

The included LUAD patients from the chemotherapy response-sensitive and -resistant groups were followed up for 3–22 months. The expression profile of miR-125b and clinical survival data of 447 LUAD patients were obtained through The Cancer Genome Atlas (TCGA) data portal (https://tcga-data.nci.nih.gov/tcga/) by the R package (version R 3.5.0). We divided all patients into the following two groups: the miR-125b low-expression group and the miR-125b high-expression group, according to the optimal cut-off point. Survival curves were plotted using the Kaplan–Meier method, and the survival was compared by the log-rank test.

### DNA constructs and transfection

The miR-125b mimics (5′-UCCCUGAGACCCUAACUUGUGA-3′), NC (5′-UUCUCCGAACGUGUCACGUTT-3′), miR-125b inhibitor (5′-UCACAA-GUUAGGGUCUCAGGGA-3′), NC-inhibitor (5′-CAGUACUUUUGUGUAGUA-CAA-3′), and siRNA duplex against human BNIP3L (sense 5′-UUCUCCGAAC-GUGUCACGUTT-3′, and antisense 5′-ACGUGACACGUUCGGAGAATT-3′), were synthesized by GenePharma (Shanghai, China). Approximately 60%-70% confluent A549/DDP cells (5 × 10^5^) were transfected with miR-125b mimics, NC, miR-125b inhibitor, NC inhibitor, or BNIP3L siRNA, at 24 h after seeding in the six-well plates, using Lipofectamine 2000 (Invitrogen, Carlsbad, CA, USA). At 48 h post-transfection, cells were harvested for further experiments.

### MTT assay

The proliferation ability of cells was detected by the MTT assay. Cells were incubated with MTT for 4 h, followed by incubation with 100 mL DMSO. The OD value at 490 nm was measured using a microplate reader (BioTek, VT, USA).

### Western blot analysis

After extracting total proteins, the concentration was determined with the BCA kit (Solaibao Technology, Beijing, China). Next, proteins were separated by the SDS-PAGE, and then electronically transferred to a polyvinylidene difluoride membrane. Following block with BSA (5%) for 1 h, the membrane was incubated with primary antibodies at 4°C overnight, including the rabbit microtubule-associated protein light chain3B (LC3B) monoclonal antibody (dilution 1:1000), rabbit Sequestosome1 (SQSTM1/P62) monoclonal antibody (dilution 1:10000), rabbit Autophagy-related protein 6 (ATG6)/beclin1 polyclonal antibody (dilution 1:1000), rabbit RORA monoclonal antibody (dilution 1:1000), rabbit BNIP3L monoclonal antibody (dilution 1:1000), rabbit P-glycoprotein (P-gp) monoclonal antibody (dilution 1:2000), rabbit Major Vault Protein (MVP) monoclonal antibody (dilution 1:2000), and rabbit β-actin monoclonal antibody (dilution 1:1000), purchased from Abcam (Boston, MA, USA). Then, the membrane was incubated with horseradish peroxidase-linked secondary antibodies for 1 h. Target proteins were detected by enhanced chemiluminescence (Zhongshi Gene Technology Co., Ltd., Tianjin, China), and the bands were scanned and quantified through the ImageJ software (MD, USA).

### mCherry-GFP-LC3B

After infection with the Ad-mCherry-GFP-LC3B adenovirus (Beyotime, Shanghai, China), the cells were subjected to methanol fixation and DAPI counterstaining, which were then observed with confocal microscopy (Olympus, Nagano, Japan). The yellow and red vesicles represented autophagosomes and autolysosomes, respectively. At least 15 images were taken from each group (magnification, ×600) and individual cells were analyzed with the ImageJ Software.

### Bioinformatics prediction

Target genes for miR-125b were predicted by the miRDB database (http://www.mirdb.org/miRDB/). The potential transcription factors that could bind to the BNIP3L promoter were predicted by the JASPAR (http://jaspar.genereg.net). Then the intersection of the miR-125b target genes and the potential transcription factors was taken by Venny (https://bioinfogp.cnb.csic.es/tools/venny/). Meanwhile, RORA transcription factor binding motifs were predicted by JASPAR. To further verify the upstream transcription factor regulation, the sequence of the BNIP3L promoter was downloaded from UCSC (http://www.genome.ucsc.edu/).

### Vector constructs and dual-luciferase reporter assay

The luciferase reporter vectors PGL3-RORA-3′ untranslated region (UTR) wild type (WT) and PGL3-RORA-3′UTR mutant (MUT) were obtained from GenePharma (Shanghai, China). Then the luciferase reporter vectors were co-transfected with miR-125b mimics or NC into the 293T cells. In a separate experiment, the promoter region p (−2000/+100) of BNIP3L cloned into a pGL-4 Basic vector and RORA overexpression construct (pcDNA3.1-RORA) were obtained from GenePharma. The 293T cells were co-transfected with BNIP3L-p (−2000/+100) Luc and pcDNA3.1-RORA. Finally, the luciferase activity was assessed by the dual-luciferase reporter system (Promega, WI, USA).

### Xenograft experiments in vivo

The A549/DDP cells were stably infected with lentivirus-NC or lentivirus-miR-125b (GenePharma, Shanghai, China). Then these cells were subcutaneously inoculated into the right flanks of 16 female BALB/c nude mice. The next day, these mice were treated intraperitoneally with 5 mg/kg DDP. Tumor size in mice was measured every 2 days. Tumor masses were excised and weighed after 28 days of inoculation. The xenograft experiment was approved by the Ethics Committee of Chengde Medical University.

### Hematoxylin-eosin (HE) and immunohistochemistry (IHC) staining

The resected tumor tissue was fixed in 4% paraformaldehyde, dehydrated, and paraffin-embedded. Thereafter, the 5-μm sections were subjected to HE staining and then observed under light microscopy. For IHC, sections were deparaffinized and incubated with primary monoclonal antibodies (1:1000 dilution), followed by secondary antibodies (1:2000 dilution). All sections were analyzed at ×400 magnification under the optical microscope (Leica, Wetzlar, Germany). The IHC analysis used multiple independent sites in the tissue samples. A total of 4–5 slides were evaluated from each animal.

### Statistical analysis

Data were represented as mean ± SD. Statistical analysis was conducted by SPSS 21.0 (SPSS, Chicago, IL, USA). Group differences were determined by Student’s *t*-test or one-way analysis of variance analysis (ANOVA). *p* < 0.05 was considered statistically significant.

## Results

### Identification of miR-125b as the DDP resistance gene of LUAD

**Figure 1:**
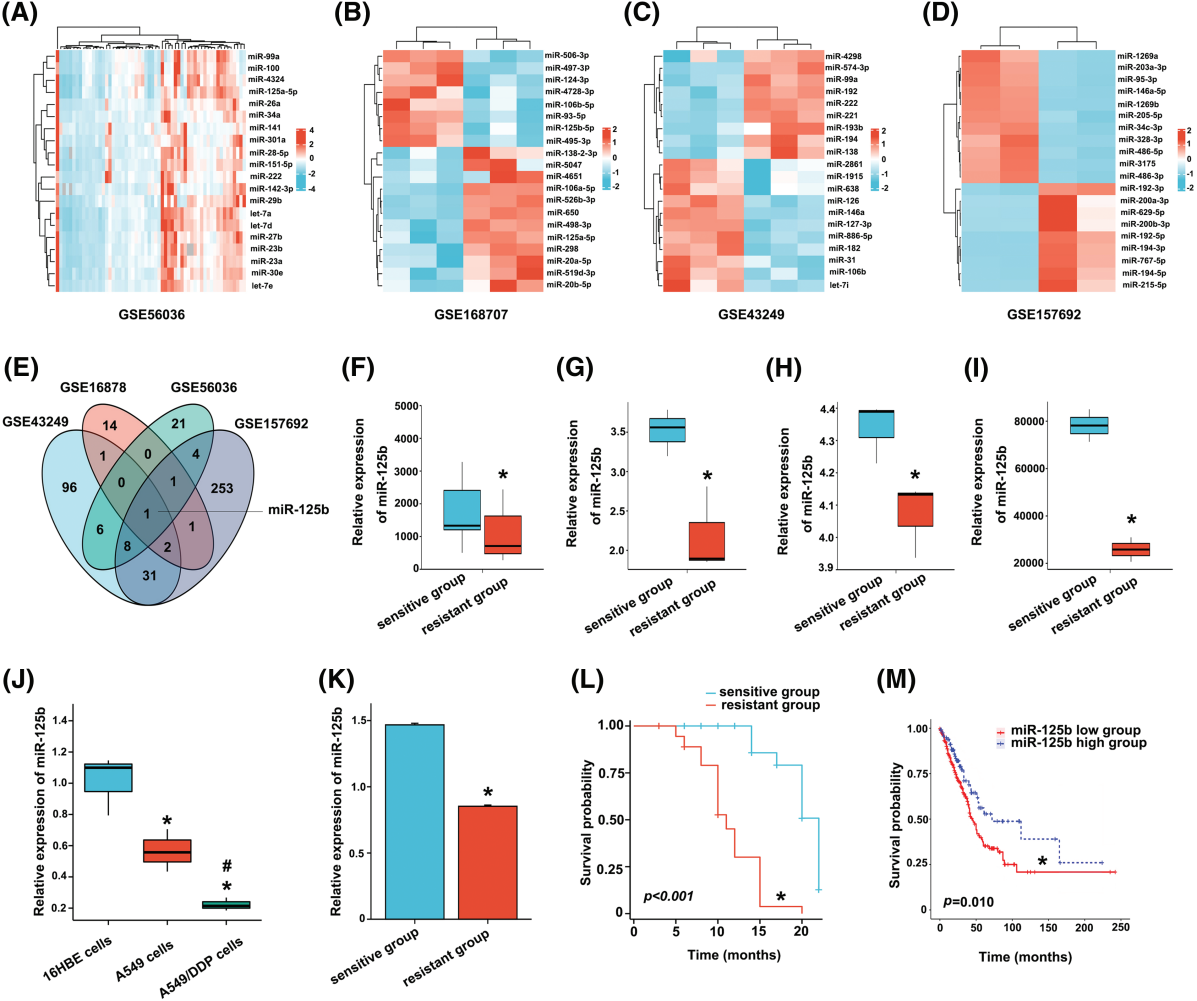
Screening and validation of the DDP-resistance related miRNAs (miR-125b) in LUAD. A-D. Visualization of differentially expressed genes. TOP20 heat map of chip GSE56036 (A), GSE168707 (B), GSE43249 (C), and GSE157692 (D); (E). Venny plot: intersection of four microarray differential genes; (F–I). MiR-125b expression of DDP-resistant and DDP-sensitive tissues/cells in chip GSE56036 (F), GSE168707 (G), GSE43249 (H), and GSE157692 (I), *p < 0.05 *vs.* sensitive group; (J), MiR-125b expression level in 16HBE, A549, A549/DDP cells detected by RT-qPCR, *p < 0.05 *vs.* 16HBE; ^#^*p* < 0.05 *vs.* A549; (K), Serum miR-125b level in the chemotherapy response-sensitive and -resistant group patients, *p < 0.05 *vs.* sensitive group; (L), Kaplan-Meier curve of 97 LUAD patients, *p < 0.05 *vs.* sensitive group; (M), Kaplan-Meier curve of 438 LUAD patients from TCGA, *p < 0.05 *vs.* miR-125b high group.

After bioinformatics analysis of two tissue and two cell DDP resistance microarray datasets extracted from the GEO database, 41 (GSE56036), 20 (GSE168707), 145 (GSE43249), and 301 (GSE157692) differentially expressed miRNAs in DDP-resistant *vs*. DDP-sensitive LUAD were identified (Figs. 1A–1D). Taking the intersection of differentially expressed miRNAs in four chips by the Venny, miR-125b was screened out (Fig. 1E). All these four GEO datasets showed that, compared to the DDP-sensitive tissues/cells, miR-125b was considerably downregulated in the DDP-resistant tissues/cells (Figs. 1F–1I). To validate the bioinformatics analysis results, the miR-125b expression in DDP-sensitive and DDP-resistant LUAD cell lines was detected by RT-qPCR. Compared with 16HBE cells, miR-125b was downregulated in both A549 (*p* = 0.008) and A549/DDP cells (*p* < 0.001, Fig. 1J). Moreover, compared with the A549 cells, the miR-125b level was lower in the A549/DDP cells (*p* = 0.023). These results suggest that miR-125b might be a DDP-resistant gene, which would be downregulated after the development of resistance to DDP.

### Low expression level of miR-125b is correlated with poor chemotherapy efficacy and prognosis in LUAD patients

A total of 55 CR and PR cases were recruited into the chemotherapy response-sensitive group, while 42 PD and SD cases were included in the chemotherapy response-resistant group. Compared with the chemotherapy response-sensitive group (1.468 ± 0.088), the serum level of miR-125b was decreased in the chemotherapy response-resistant group (0.088 ± 0.059, *p* < 0.001, Fig. 1K). Subsequently, a 22-month follow-up was conducted. Overall survival estimates showed that patients in the chemotherapy response-resistant group had obviously shorter survival than those in the chemotherapy response-sensitive group (Fig. 1L, *p* < 0.001).

Similar results were obtained in the TCGA data analysis. The cut-off value (cut-off value = 87) was calculated based on the expression level of miR-125b in LUAD from TCGA, and 438 patients were divided into the following two subgroups: one group with a high level of miR-125b above the cut-off value (118 patients), and the other group with a low level of miR-125b beneath the cut-off value. Combined with TCGA survival data, the survival analysis was performed with the Kaplan-Meier curves, and the results revealed that the overall survival of patients in the low miR-125b expression group was lower than the high miR-125b expression group (Fig. 1M, *p* = 0.010).

### miR-125b reverses DDP resistance in A549/DDP cells

**Figure 2:**
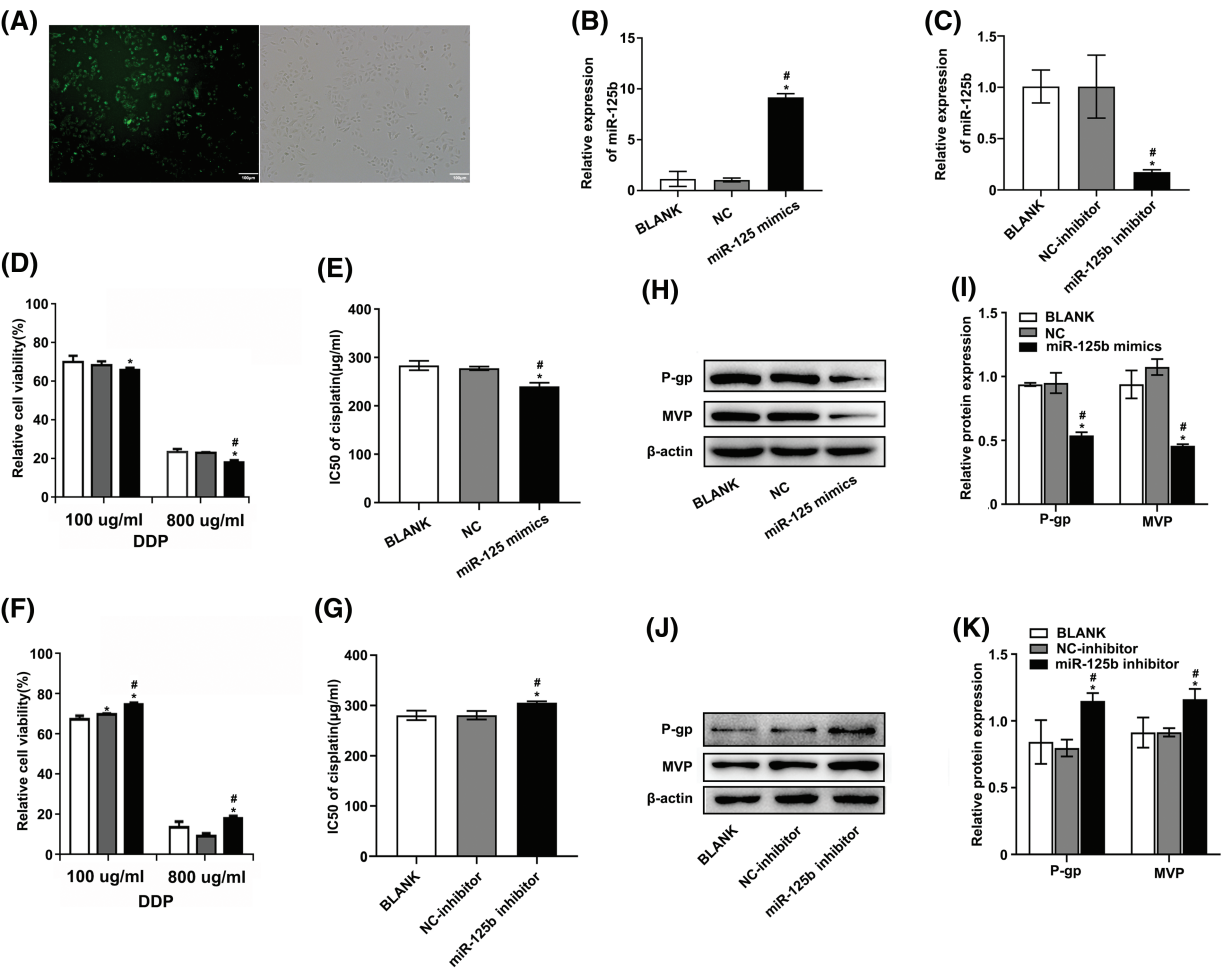
miR-125b reverses the DDP resistance in A549/DDP cells. (A). The transient transfection efficiency of miR-125b; (B and C). MiR-125b levels in A549/DDP cells after transfection with NC/miR-125b mimics (B) or NC-inhibitor/miR-125b inhibitor (C) were detected via RT-qPCR assays; (D and F). Cell viability is detected using the MTT assay in A549/DDP cells transfected with NC/miR-125b mimics (D) or NC-inhibitor/miR-125b inhibitor (F) followed by the DDP treatment with the concentration gradient; (E and G). IC50 values of DDP in A549/DDP cells transfected with NC/miR-125b mimics (E) or NC-inhibitor/miR-125b inhibitor (G); (H–K). The expression levels of MDR‑related proteins in A549/DDP cells transfected with NC/miR-125b mimics (H and I) or NC-inhibitor/miR-125b inhibitor (J and K) are detected via Western blot assays. *p < 0.05 *vs.* BLANK group; ^#^*p* < 0.05 *vs.* NC/NC-inhibitor group.

The A549/DDP cells were transfected with miR-125b mimics or inhibitors. Meanwhile, NC and NC inhibitors were transfected as control. Additionally, NC labeled with 5-FAM (green) was also transfected and monitored with a fluorescence microscope. The transfection efficiency was 83.12 ± 8.41% as compared with the mock-transfected cells (Fig. 2A). Furthermore, compared to cells transfected with NC, the miR-125b levels were considerably higher in cells transfected with miR-125b mimics (Fig. 2B, *p* < 0.001). Conversely, the levels of miR-125b were lower in cells transfected with miR-125b inhibitors than those transfected with NC inhibitors (Fig. 2C, *p* = 0.002). These results suggested the successful construction of the A549/DDP cells overexpressing or downregulating miR-125b.

To determine how miR-125b affected the growth of DDP-resistant cells, the cell viability was detected using the MTT assay, at 48 h after transfection. Under various concentrations of DDP, the relative proliferation rates of A549/DDP cells after miR-125b mimic transfection were 85.58 ± 1.13%, 75.78 ± 2.42%, 64.02 ± 1.08%, 56.91 ± 1.54%, 49.11 ± 0.63%, and 17.59 ± 1.14%, respectively. While the relative proliferation rates of the cells transfected with NC were 88.22 ± 1.92%, 78.23 ± 0.96%, 66.47 ± 1.92%, 56.82 ± 0.52%, 46.85 ± 1.37%, and 22.22 ± 0.49%, respectively (Fig. 2D). Compared to the NC group, the 50% inhibitory concentration (IC50) value of the miR-125b group was significantly decreased (*p* = 0.001, Fig. 2E). Conversely, after the miR-125b inhibitor transfection, the relative proliferation rates were 93.32 ± 1.72%, 81.86 ± 1.83%, 76.29 ± 1.14%, 73.77 ± 1.16%, 48.24 ± 1.51%, and 18.67 ± 1.14%, under various concentrations of DDP. While the relative proliferation rates of the cells transfected with NC inhibitor were 90.83 ± 1.49%, 78.45 ± 1.43%, 71.51 ± 0.41%, 68.99 ± 1.32%, 47.79 ± 3.08%, and 9.36 ± 1.59%, respectively (Fig. 2F). Compared to the NC inhibitor group, the IC50 value was obviously increased in the miR-125b inhibitor group (*p* = 0.006, Fig. 2G). These findings demonstrate that miR-125b overexpression would dramatically reduce the A549/DDP cell proliferation while the miR-125b downregulation would increase cell proliferation.

To explore whether miR-125b altered multidrug-resistant (MDR)-related genes, the Western blot was employed to determine the expression levels of P-gp [13] and MVP [14], at 48 h after being transfected with miR-125b mimics/inhibitor. Compared with NC, the A549/DDP cells with miR-125b mimics showed lower protein expression levels of P-gp (*p* < 0.001) and MVP (*p* < 0.001, Figs. 2H and 2I). On the contrary, the expression levels of P-gp (*p* < 0.001) and MVP (*p* = 0.001) in the A549/DDP cells with miR-125b inhibitor were higher than the NC inhibitor (Figs. 2J and 2K).

Taken together, miR-125b attenuated the cell proliferation and the protein expression of MDR-related genes in A549/DDP cells, which suggests that miR-125b reverses the NSCLC cells’ resistance to DDP.

### miR-125b inhibits autophagy in A549/DDP cells

**Figure 3:**
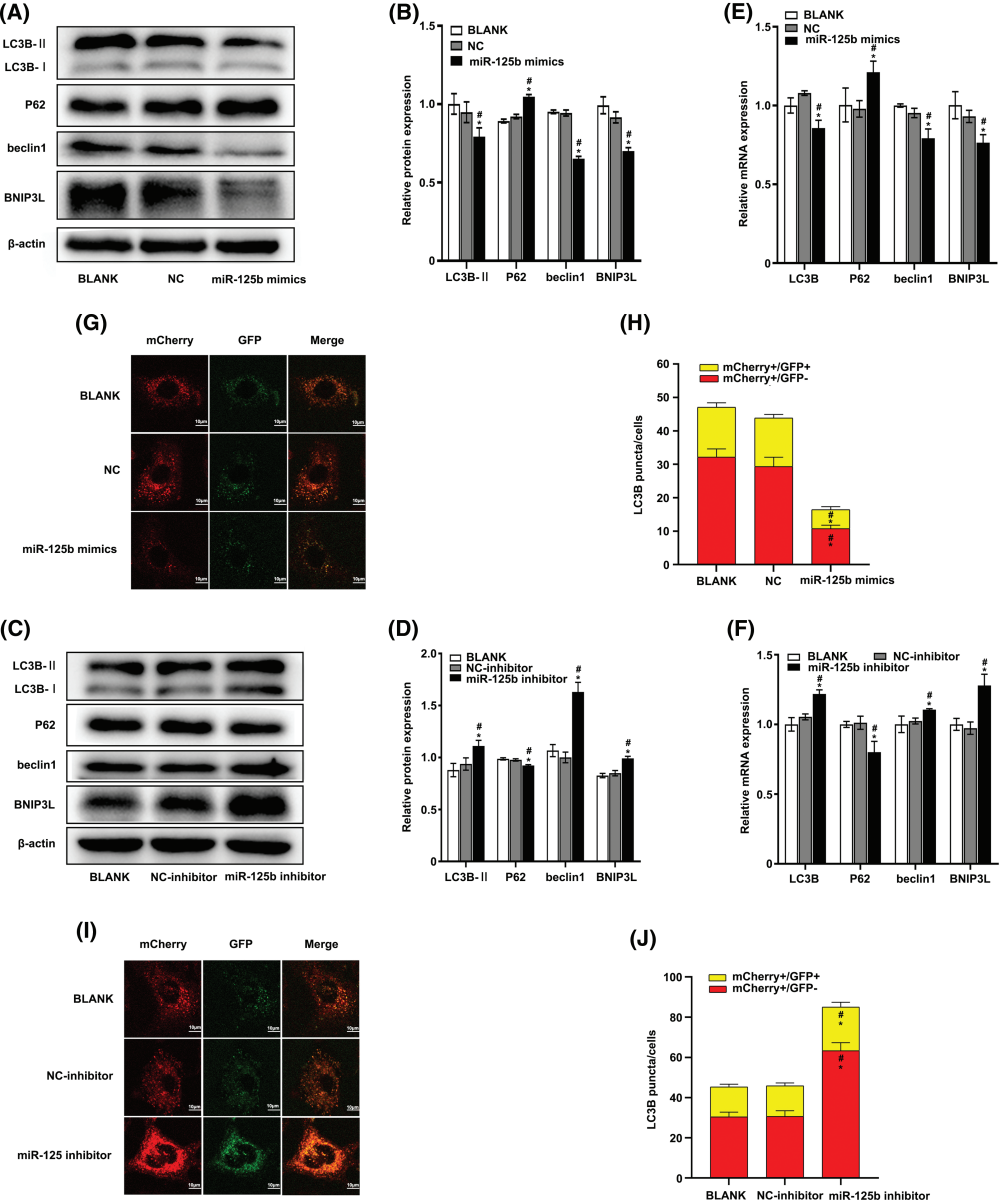
miR-125b inhibits autophagy in A549/DDP cells (A–F). The expression level of autophagy-related genes after transfection with NC/miR-125b mimics or NC-inhibitor/miR-125b inhibitor are detected by Western blot (A–D) and RT-qPCR assays (E and F); (G and I). Representative immunofluorescence images of the Ad-GFP-mCherry-LC3B in A549/DDP cells with NC/miR-125b mimics (G) or NC-inhibitor/miR-125b inhibitor (I); (H and J). Numbers of the yellow and red fluorescent puncta in A549/DDP cells with NC/miR-125b mimics (H) or NC-inhibitor/miR-125b inhibitor (J) are calculated. *p < 0.05 *vs.* BLANK group; ^#^*p* < 0.05 *vs.* NC/NC-inhibitor group.

At 48 h post-transfection, the total protein extract was analyzed for autophagy markers using the Western blot method. The results showed that the expression levels of LC3B-II (*p* = 0.023), beclin-1 (*p* < 0.001), and BNIP3L (*p* = 0.001) were significantly decreased, while the p62 expression levels (*p* < 0.001) were obviously increased in the miR-125b mimics group compared with the NC group (Figs. 3A and 3B). In contrast, compared with the NC inhibitor group, the expression levels of LC3B-II (*p* < 0.001), beclin-1 (*p* < 0.001), and BNIP3L (*p* < 0.001) were significantly higher, while the p62 protein expression levels (*p* = 0.001) were obviously lower, in the miR-125b inhibitor group (Figs. 3C and 3D).

At 24 h after transfection, the RT-qPCR was performed to determine the effect of miR-125b on the autophagy markers. The results showed that the mRNA levels of LC3B (*p* = 0.001), beclin-1 (*p* = 0.002), and BNIP3L (*p* = 0.016) were lower, while the mRNA expression level of P62 (*p* = 0.012) was higher in cells with miR-125b mimics, compared with the cells with NC (Fig. 3E). Conversely, compared with the NC inhibitor group, the mRNA levels of LC3B (*p* = 0.001), beclin-1 (*p* = 0.035), and BNIP3L (*p* = 0.001) were higher, and the P62 mRNA levels (*p* = 0.003) were lower, in the miR-125b inhibitor group (Fig. 3F).

To further verify whether miR-125b influenced autophagic flux, the miR-125b mimics/inhibitor and ad-mCherry-GFP-LC3B adenoviruses were transfected into the A549/DDP cells. Autophagosome and autophagolysosome puncta were rendered in yellow and red, respectively, under laser confocal microscopy. Compared to the NC group, both the autophagosome (mCherry+/GFP+, *p* < 0.001) and autophagolysosome puncta (mCherry+/GFP−, *P* < 0.001) were decreased in the miR-125b mimics group (Figs. 3G and 3H). Conversely, both the autophagosome (*p* = 0.012) and autophagolysosome puncta (*p* < 0.001) were considerably higher in the miR-125b inhibitor group than those in the NC inhibitor group (Figs. 3I and 3J). These data confirm that miR-125b reduces autophagosome, thereby inhibiting autophagy. Altogether, miR-125b inhibited both transcript and translation levels of autophagy-related genes, and the formation of autophagosome, suggesting that miR-125b can suppress autophagy.

### miR-125b reverses DDP resistance by inhibiting autophagy

**Figure 4:**
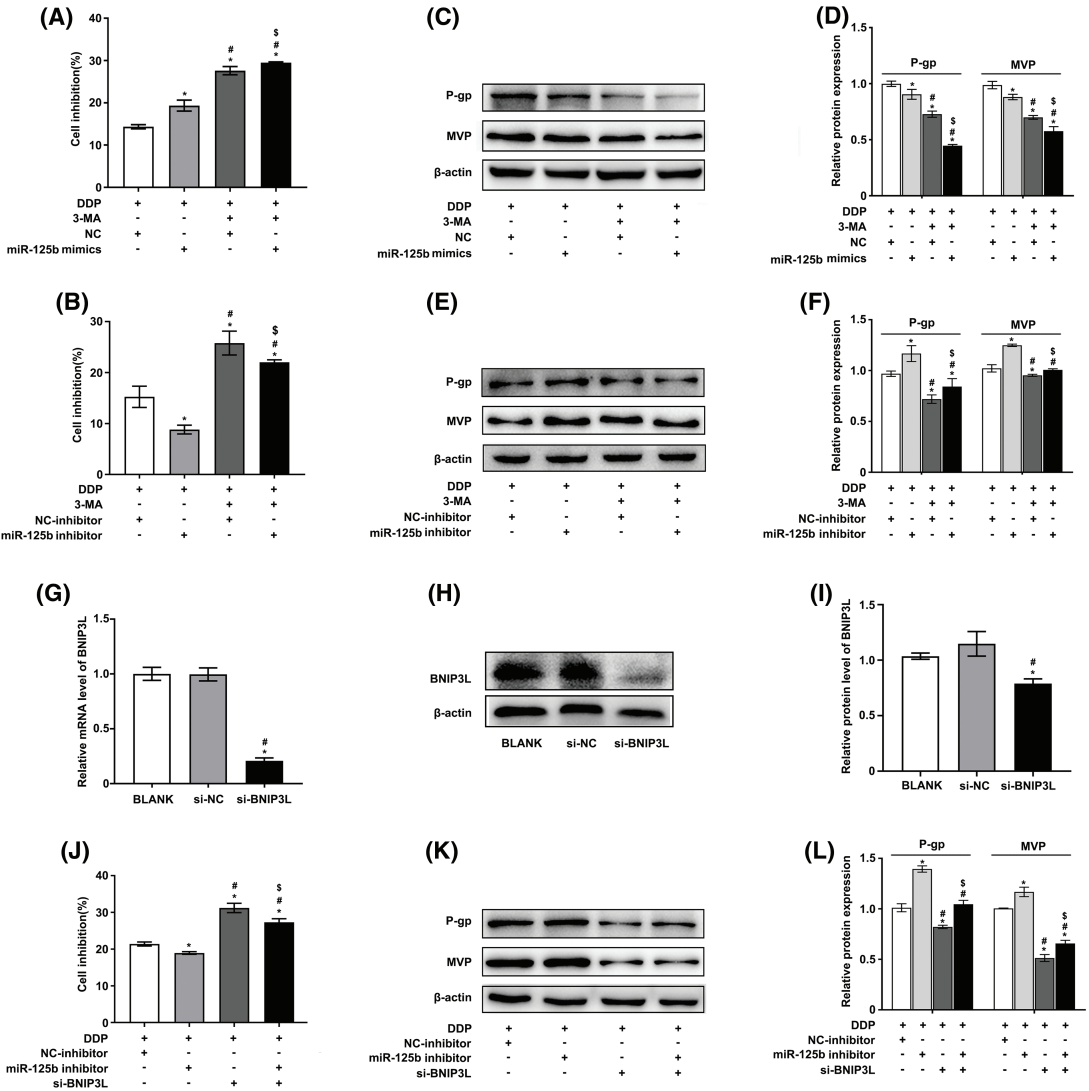
miR-125b reverses DDP resistance via inhibiting autophagy. (A–F). A549/DDP cells transfected with NC/miR-125b mimics or NC-inhibitor/miR-125b inhibitor are treated with 3-MA (5 mM) and exposed to DDP (27.8 μg/ml), then cell inhibition is measured by the MTT assays (A and B). Meanwhile, the protein expressions of MDR‑related genes are detected by Western blot (C–F). *p < 0.05 *vs.* NC/ NC-inhibitor group; ^#^*p* < 0.05 *vs.* miR-125b mimics/ miR-125b inhibitor group; ^$^*p* < 0.05 *vs.* NC/ NC-inhibitor +3-MA group (G–I). The efficiency of transfection is validated by RT-qPCR (G) and Western blot assays (H and I) in BNIP3L-overexpressed A549/DDP cells. *p < 0.05 *vs.* BLANK group; ^#^*p* < 0.05 *vs.* si-NC group (J–L). A549/DDP cells transfected with NC-inhibitor/miR-125b inhibitor and si-BNIP3L, are exposed to DDP (27.8 μg/ml), then cell inhibition is measured by the MTT assays (J). Meanwhile, the protein expressions of MDR‑related genes are detected by Western blot (K and L). *p < 0.05 *vs.* NC-inhibitor group; ^#^*p* < 0.05 *vs.* miR-125b inhibitor group; ^$^*p* < 0.05 *vs.* si-BNIP3L group.

Whether miR-125b modulated the DDP resistance of LUAD cells by regulating autophagy was then explored. The results showed that 3-methyladenine (3-MA), an autophagy inhibitor, promoted the ability of DDP to suppress cell viability, and miR-125b mimics could further enhance the phenotype, while miR-125b inhibitor reversed this effect, in A549/DDP cells (Figs. 4A and 4B). Furthermore, Western blot was used to examine the expression levels of MDR-related genes. The results showed that 3-MA downregulated the P-gp and MVP protein levels, and miR-125b mimics further reduced the levels (Figs. 4C and 4D), while miR-125b inhibitor partially reverted the effects (Figs. 4E and 4F).

To further verify these findings, autophagy was suppressed by knocking down the autophagic inducer BNIP3L. The knocking-down efficiency was identified by RT-qPCR (Fig. 4G) as well as Western blot analysis (Figs. 4H and 4I). The results showed similar results as described above. Silencing BNIP3L induced the increase in cell inhibition rates, and however, the effect would be partially restored by miR-125b inhibitor, in A549/DDP cells (Fig. 4J). Additionally, BNIP3L knocking down largely suppressed the P-gp and MVP expression, while the miR-125b inhibitor partially reverted these effects (Figs. 4K and 4L). The rescuing experiment was performed using the autophagy inhibitor or silencing BNIP3L, suggesting that miR-125b reverses DDP resistance by inhibiting autophagy.

### RORA/BNIP3L axis is identified as a potential target of miR-125b

**Figure 5:**
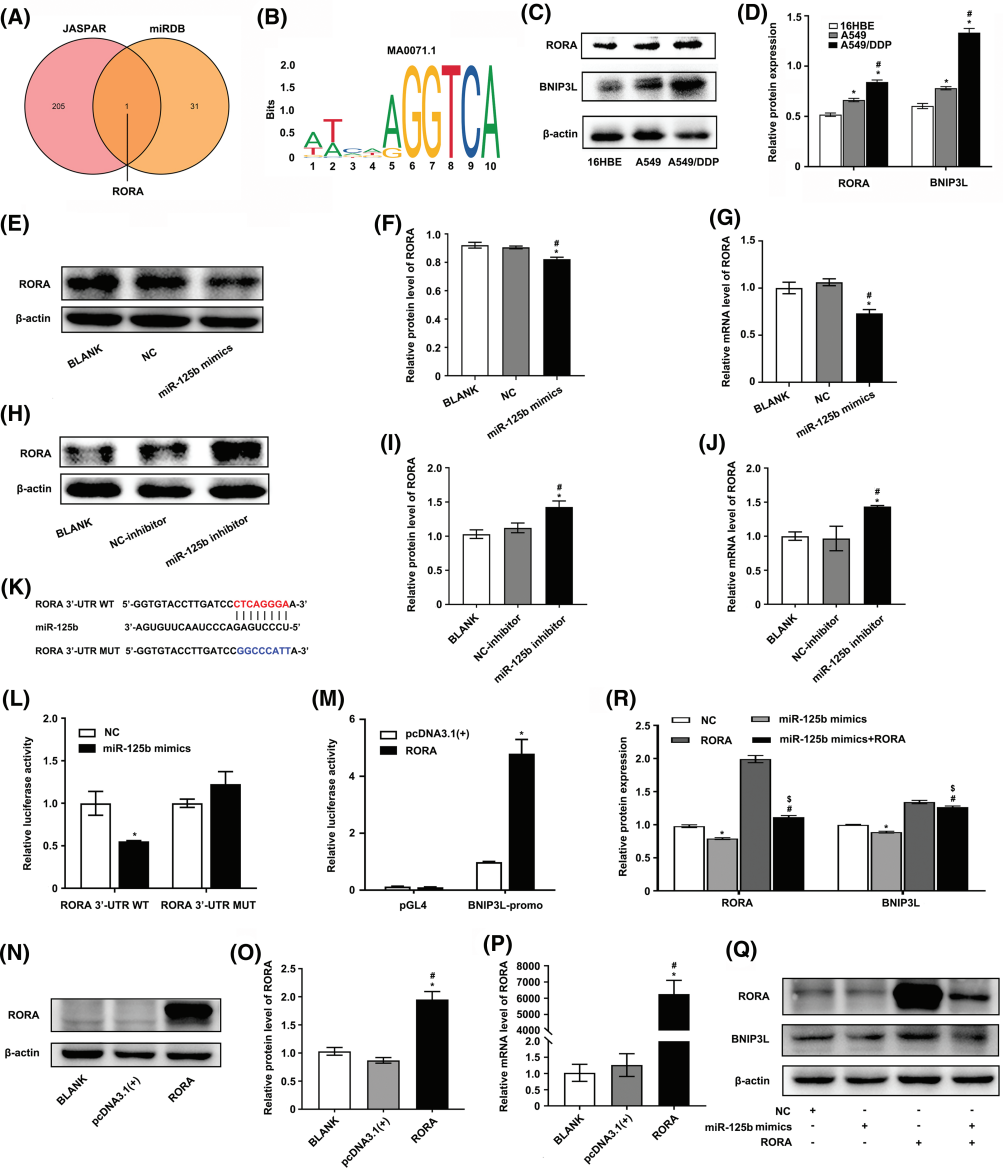
RORA/BNIP3L axis is identified as a potential target of miR-125b (A). Venny shows the intersection of miRDB and JASPAR database; (B). The motif of transcription factor RORA; (C and D). Protein expressions of RORA and BNIP3L in 16HBE, A549, and A549/DDP cell lines are assayed by Western blot; (E–J). The effects of NC/miR-125b mimics or NC-inhibitor/miR-125b inhibitor on RORA expression levels are determined by Western blot (E, F, H and I), as well as qRT-PCR assays (G and J). *p < 0.05 *vs.* BLANK group; ^#^*p* < 0.05 *vs.* NC/NC-inhibitor group; (K). The binding site between miR-125b and the 3′UTR of RORA; (L). A dual luciferase reporter assay is implemented to certify the miR-125b-RORA association. *p < 0.05 *vs.* NC group; (M). A dual luciferase reporter assay is implemented to certify the RORA-BNIP3L association. *p < 0.05 *vs.* pcDNA3.1(+) group; (N–P). The efficiency of transfection is validated by Western blot (N and O) and RT-qPCR assays (P). *p < 0.05 *vs.* BLANK group; ^#^*p* < 0.05 *vs.* pcDNA3.1(+) group (Q and R). A549/DDP cells are divided into the NC, mimics, RORA, and mimics+RORA groups, and the protein levels of RORA and BNIP3L are assayed by Western blot. *p < 0.05 *vs.* NC group; ^#^*p* < 0.05 *vs.* miR-125b mimics group; ^$^*p* < 0.05 *vs.* RORA group.

**Figure S1:**
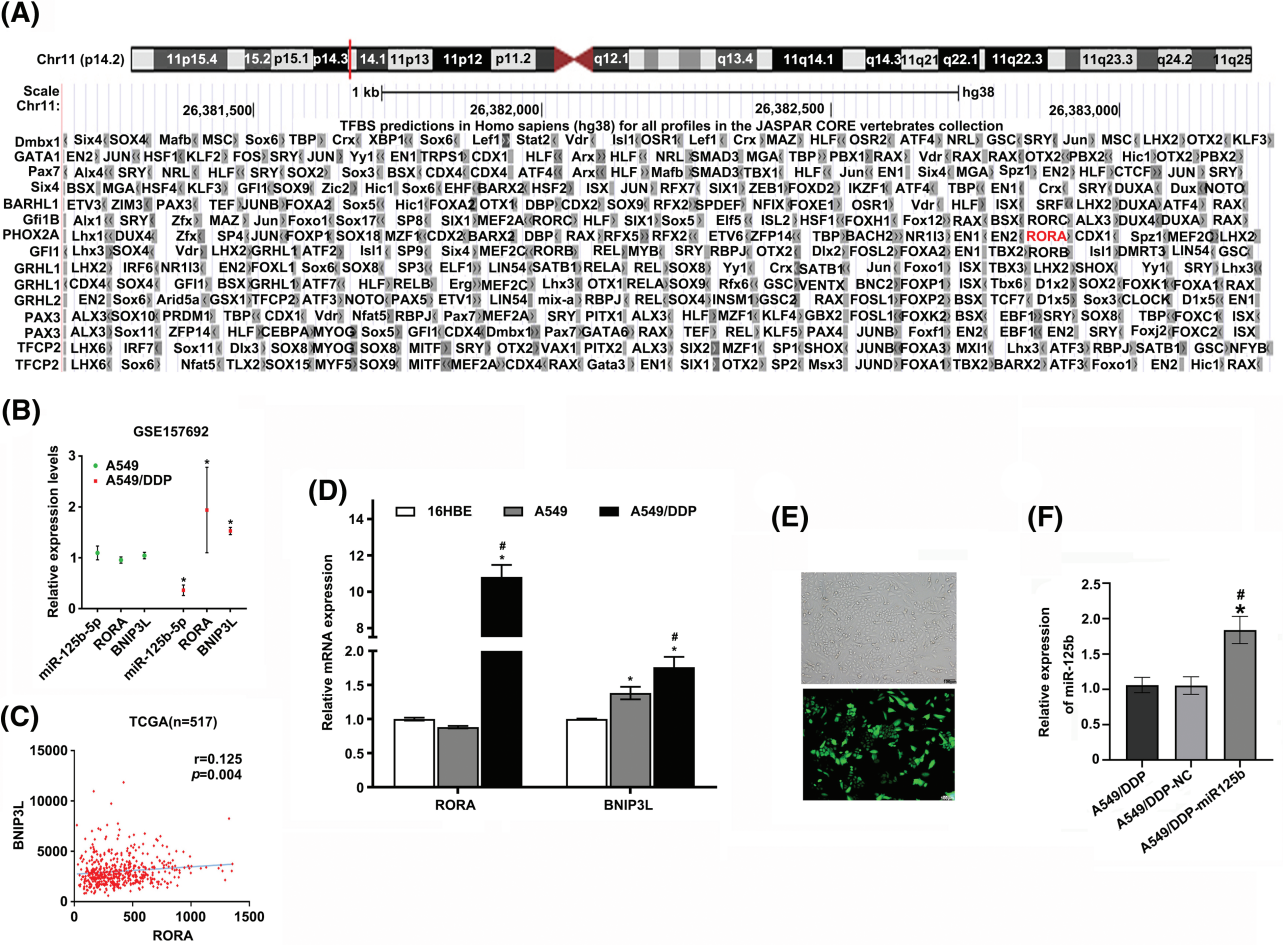
(A). The predicted result of UCSC website; (B). Relative expression levels of miR-125b/RORA/BNIP3L in A549 and A549/DDP cells in GSE157692. *p < 0.05 *vs.* A549 group; (C). Correlation of RORA and BNIP3L expression levels in 517 patients with LUAD in TCGA database (r = 0.125, *p* = 0.004); (D). The mRNA expression levels of RORA/BNIP3L in 16HBE, A549, and A549/DDP cell lines are detected by RT-qPCR assays. *p < 0.05 *vs.* 16HBE group; ^#^*p* < 0.05 *vs.* A549 group; (E). The infection efficiency of miR-125b overexpression lentivirus vector; (F). Expression levels of miR-125b in A549/DDP cells after infection with miR-125b overexpression lentivirus vector are detected using RT-qPCR assays; *p < 0.05 *vs.* A549/DDP-NC; ^#^*p* < 0.05 *vs.* A549/DDP.

To explore how miR-125b regulated autophagy, the candidate targets of miR-125b were predicted by the miRDB, and the transcription factors binding to BNIP3L promoter were predicted by the JASPAR. Finally, RORA was obtained by taking the intersection of the 32 target genes and the 206 transcription factor genes (Fig. 5A). The DNA motif of the RORA transcription factor was downloaded from JASPAR (Fig. 5B). Further, the results validated that RORA was a potential transcription factor candidate that might interact with the BNIP3L promoter region through browsing UCSC (Track score 302, *p* < 0.001, Suppl. Fig. S1A).

Next, the expression profile of miR-125b, RORA, and BNIP3L in LUAD patients and DDP-resistant cells was analyzed using the raw gene expression data (GSE157692) downloaded from GEO. The results demonstrated that the miR-125b (*p* = 0.026) expression level was decreased, while the RORA (*p* = 0.046) and BNIP3L (*p* = 0.019) levels were increased in the A549/DDP cells, compared with the A549 cells (Suppl. Fig. S1B). A positive correlation between RORA and BNIP3L expression (*p* = 0.004) was observed by analyzing the normalized expression data of RORA and BNIP3L downloaded from the TCGA (Suppl. Fig. S1C). Furthermore, the Western blot analysis showed that the RORA (*p* < 0.001) and BNIP3L (*p* < 0.001) expressions were elevated in the A549/DDP cells, compared with the A549 cells (Figs. 5C and 5D). Similar results were obtained with the RT-qPCR analysis. Compared with the A549 cells, the mRNA levels of RORA (*p* < 0.001) and BNIP3L (*p* = 0.004) were significantly increased in the A549/DDP cells (Suppl. Fig. S1D), while the mRNA expression levels of miR-125b (*p* = 0.023) showed a significant decrease (Fig. 1J).

To determine the upstream and downstream regulation relationship of miR-125b and RORA/BNIP3L, the RORA and BNIP3L levels were measured by the RT-qPCR and Western blot after transfection with miR-125b mimics or miR-125b inhibitor. The results demonstrated that miR-125b mimics decreased the mRNA (*p* < 0.001) and protein expression levels (*p* = 0.001) of RORA (Figs. 5E–5G) and BNIP3L (Figs. 3A and 3B), while miR-125b inhibitor increased the mRNA (*p* = 0.002) and protein (*p* = 0.002) expression levels of RORA (Figs. 5H–5J) and BNIP3L (Figs. 3C and 3D), suggesting that miR-125b regulates the expression levels of RORA and BNIP3L. Thus, we propose that RORA, as a transcription factor for the autophagic inducer BNIP3L, is a potential target of miR-125b.

To further confirm the regulating effects of the miR-125b/RORA/BNIP3L axis, the luciferase reporters for the wild-type (RORA-3′-UTR-WT) and mutated RORA’s 3′UTR (RORA-3′-UTR-MUT) were constructed (Fig. 5K). Then, the luciferase reporter assay was performed in the 293T cells that had been transfected with NC or miR-125b mimics. The WT RORA 3′UTR reporter was strongly suppressed by increased miR-125b, rather than the mutant RORA 3′UTR reporter that lacked the miR-125b binding site (*p* = 0.001, Fig. 5L). Therefore, miR-125b might target RORA directly *in vitro*. Next, the putative 2.1-kb BNIP3L promoter sequence (−2.0/+0.1 kb) was cloned into the pGL4 firefly luciferase reporter vector to determine whether RORA controlled transcription of BNIP3L through binding to its promoter. The 293T cells were transfected with this promoter-reporter and RORA expression constructs or control vector (pcDNA3.1). As shown in Fig. 5M, after 36 h, the dual luciferase reporter assay revealed that RORA remarkably activated transcription from the BNIP3L promoter (*p* = 0.017), which was not changed in the control groups (*p* = 0.084).

Furthermore, RT-qPCR and Western blot were conducted to confirm the RORA overexpression following transfection in A549/DDP cells (Figs. 5N–5P). Next, the A549/DDP cells were transfected with RORA plasmid, miR-125b mimics, or co-transfected of both, respectively. Western blot analysis showed that the RORA (*p* < 0.001) and BNIP3L (*p* < 0.001) protein levels were significantly increased in the cells transfected with RORA plasmid, however, both of them were significantly suppressed in the cells co-transfected with RORA plasmid and miR-125b mimics (RORA: *p* < 0.001; BNIP3L: *p* = 0.001; Figs. 5Q and 5R). These findings indicate that the BNIP3L expression level would be elevated with overexpression of RORA, and however, when the RORA expression is suppressed by miR-125b mimics, there would be a concomitant suppression of BNIP3L. Taken together, in the A549/DDP cells, there is a direct regulation of the miR-125b/RORA/BNIP3L axis. Specifically, miR-125b binds to the 3′ UTR of the RORA mRNA and negatively regulates RORA expression. RORA, in turn, as a transcription factor, influences the expression of BNIP3L.

### miR-125b regulates autophagy and reverses resistance by targeting RORA/BNIP3L

**Figure 6:**
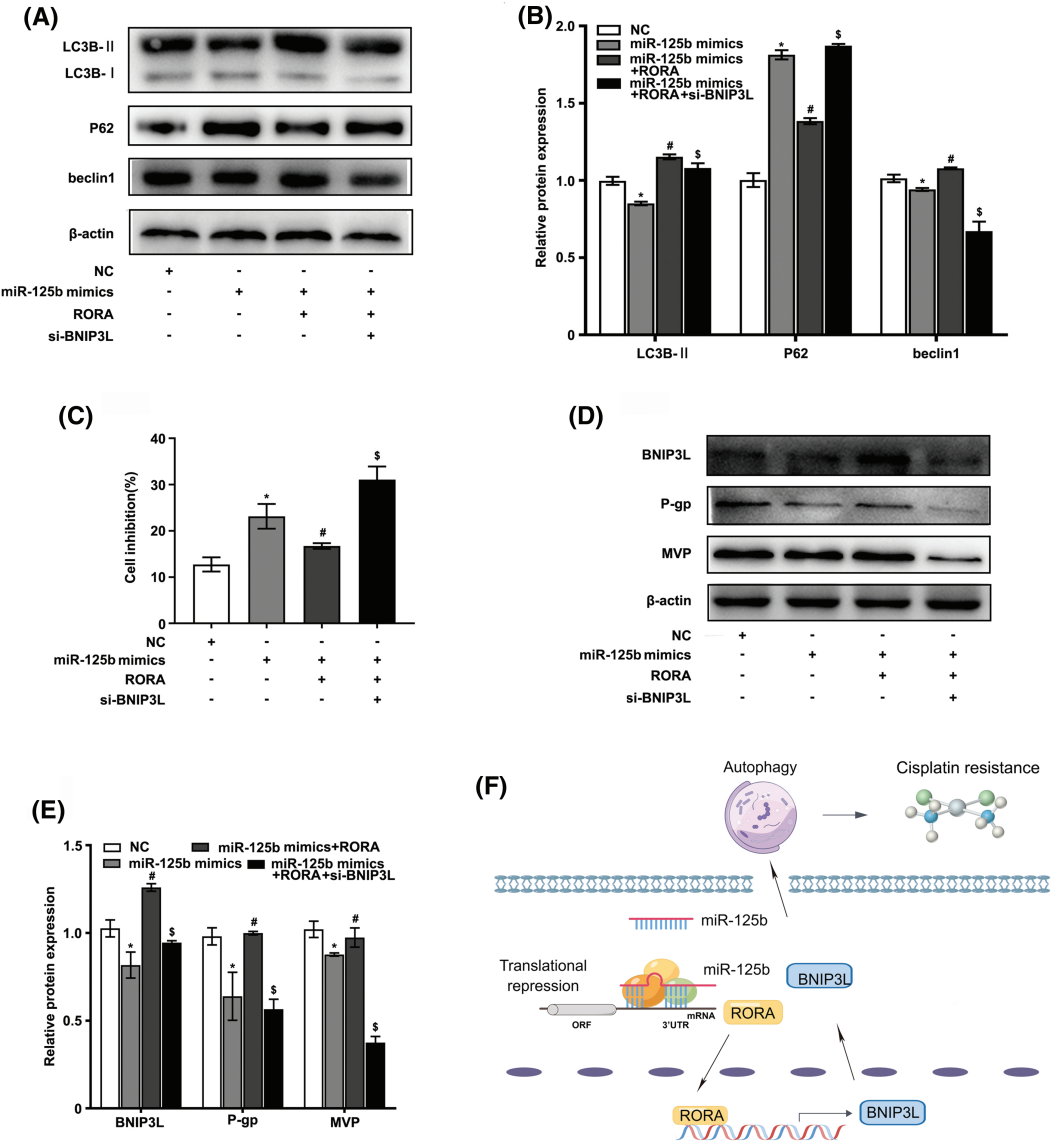
miR-125b/RORA/BNIP3L axis reverses DDP resistance by regulating autophagy. A549/DDP cells are divided into the NC, miR-125 mimics, miR-125b mimics+RORA, and miR-125b mimics+RORA+si-BNIP3L groups. (A and B). The protein levels of autophagy-related genes in groups are assayed by Western blot; (C). Cell inhibition with DDP (27.8 μg/ml) is detected by MTT assays; (D and E). The expression levels of BNIP3L and MDR-related genes are detected using Western blot assays. *p < 0.05 *vs.* NC group; ^#^*p* < 0.05 *vs.* miR-125b mimics group; ^$^*p* < 0.05 *vs.* miR-125b mimics+RORA group. (F). Diagram of molecular mechanism.

To determine whether miR-125b regulated autophagy by targeting RORA/BNIP3L, subsequently reversing resistance, the A549/DDP cells were co-transfected with miR-125b mimics, RORA plasmid, and si-BNIP3L, or co-transfected with miR-125b mimics and RORA plasmid, or transfected with miR-125b mimics alone. First, the expression levels of autophagy markers were detected using the Western blot analysis. As shown in Figs. 6A and 6B, the expression levels of LC3B-II and beclin1 were decreased in the A549/DDP cells transfected with miR-125b mimics alone compared with the NC group (LC3B-II: *p* < 0.001; beclin1: *p* = 0.033), which were increased in cells co-transfected with miR-125b mimics and RORA plasmid compared with miR-125b mimics-transfected cells (LC3B-II: *P* < 0.001; beclin1: *p* = 0.001), and decreased in cells co-transfected with miR-125b mimics, RORA plasmid, and si-BNIP3L (LC3B-II, beclin1: *p* < 0.001) compared with the miR-125b mimics and RORA co-transfection group. The p62 protein expression levels in each group exhibited the exact opposite pattern (mimics: *p* < 0.001; mimics+RORA: *p* < 0.001; mim-ics+RORA+si-BNIP3L: *p* = 0.042). The above results show that miR-125b mimics inhibit autophagy in A549/DDP, and the autophagy is enhanced after co-treatment of miR-125b mimics and RORA plasmid. Finally, autophagy is inhibited again by a combination of miR-125b mimics, RORA plasmid, and si-BNIP3L, which means miR-125b regulates autophagy through RORA/BNIP3L.

Next, the cell viability in various groups was detected by the MTT assay. As shown in Fig. 6C, compared with the NC group, the cell growth inhibition rate was drastically increased in the miR-125 mimics group (*p* < 0.001), which was decreased in the miR-125b mimics and RORA co-transfection cells compared with miR-125 mimics group (*p* = 0.006), and increased again in cells co-treated with miR-125b mimics, RORA plasmid, and si-BNIP3L compared with the miR-125b mimics and RORA co-transfection group (*p* < 0.001).

Furthermore, the protein expressions of MDR-related genes were monitored by the Western blot analysis (Figs. 6D and 6E). The g-gp (*p* = 0.001) and MVP (*p* = 0.002) levels were lower in the miR-125b mimics group than in the NC group. Compared with the miR-125b mimics group, the p-gp (*p* < 0.001) and MVP (*p* = 0.018) protein levels were significantly increased in the miR-125b mimics and RORA co-transfection groups. The p-gp (*p* < 0.001) and MVP (*p* < 0.001) levels were suppressed again after co-transfection with miR-125b mimics, RORA plasmid, and si-BNIP3L, compared with the miR-125b mimics and RORA co-transfection group. These results suggest that miR-125b might reverse the A549/DDP cells’ resistance to DDP, and the drug resistance-reversing effect would be reduced by the combination of miR-125b mimics and RORA plasmid, whereas the effect is partially restored after co-treated with miR-125b mimics, RORA plasmid, and si-BNIP3L. In summary, all these findings indicate that miR-125b regulates autophagy, and in turn, reverses resistance by directly targeting RORA/BNIP3L (Fig. 6F).

### miR-125b inhibits DDP-resistant tumor growth and autophagy *in vivo*

**Figure 7:**
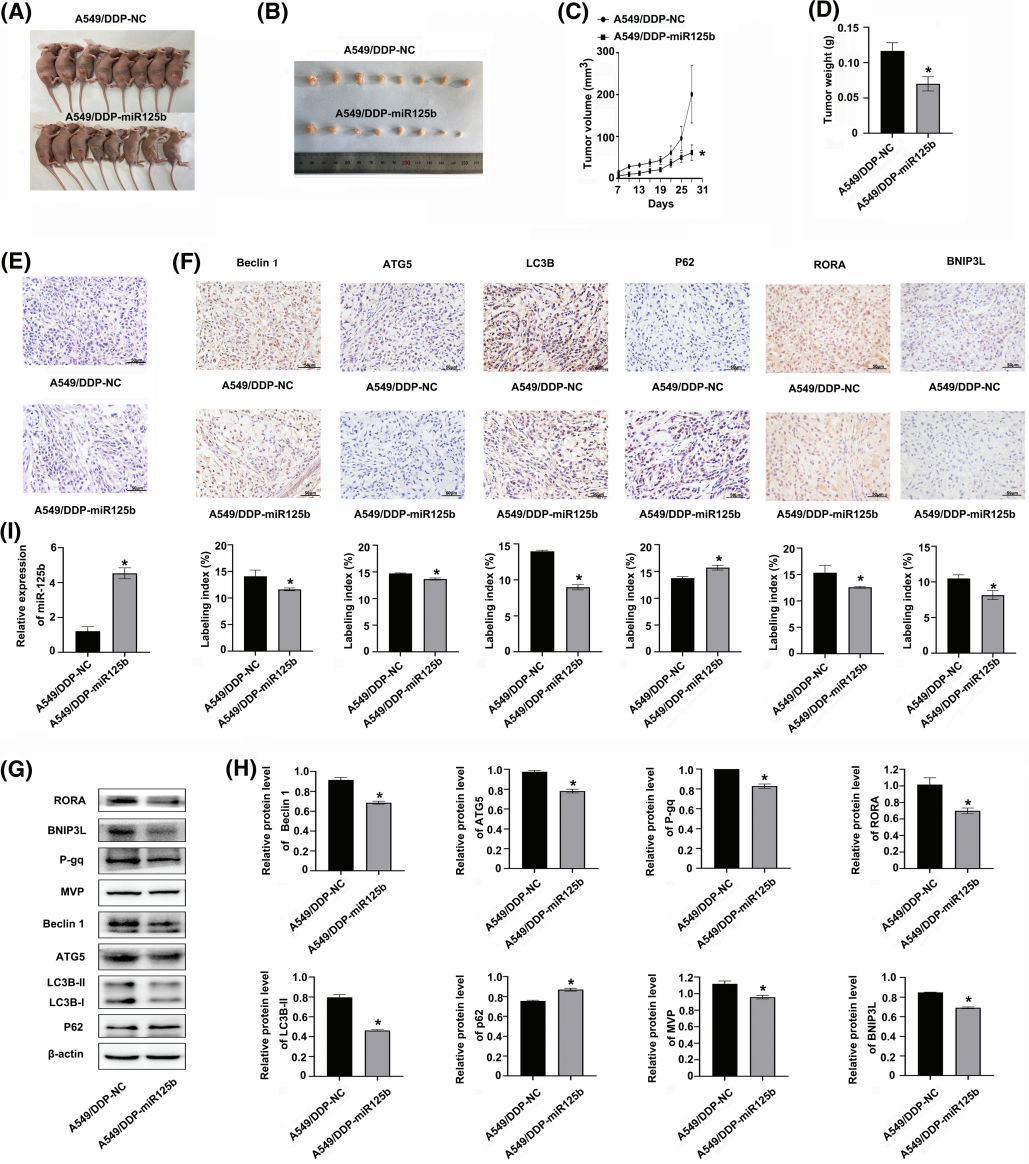
miR-125b inhibits DDP-resistant tumor growth by regulating autophagy and RORA/BNIP3L axis *in vivo* (A). Image of nude mice with subcutaneous xenograft tumor; (B). Image of resected xenograft tumor; (C). Growth curves of subcutaneous xenograft tumor size; (D). Tumor xenograft weight; (E). HE staining, magnification &times; 400; (F). Protein expressions of autophagy-related genes and RORA/BNIP3L are detected by IHC, magnification &times; 400; (G and H). Protein expressions of RORA/BNIP3L, MDR- and autophagy-related are assayed by Western blot; (I). miR125b expressions in xenograft tumors are detected by RT-qPCR. *p < 0.05 *vs.* A549/DDP-NC; ^#^*p* < 0.05 *vs.* A549/DDP.

The A549/DDP cells were infected with the miR-125b overexpression lentivirus vector, and the infection efficiency was 85.25 ± 3.92% (Suppl. Fig. S1E). Meanwhile, the miR-125b expression levels were upregulated in cells at 3 d after infection of miR-125b overexpression lentivirus vector (A549/DDP-miR125b), compared with the non-infected control cells (A549/DDP, *p* = 0.002) or cells infected with the control vector (A549/DDP-NC, *P* = 0.001, Suppl. Fig. S1F). Next, the A549/DDP-miR125b cells and the A549/DDP-NC cells were injected subcutaneously into the nude mice, respectively, and the both two group mice were treated with DDP (5 mg/kg) at the same time. The results demonstrated that miR-125b inhibited the subcutaneous xenograft tumor growth (Figs. 7A and 7B). Compared with the A549/DDP-NC group, the tumor weight (*p* = 0.001) and volume (*p* = 0.049) were decreased in the A549/DDP-miR125b group (Figs. 7C and 7D). Moreover, HE staining revealed that tumors formed by cells infected with miR-125b showed less malignancy (Fig. 7E).

To determine whether miR-125b regulated autophagy *in vivo*, the expression status of autophagy marker proteins was examined by IHC and Western blot analysis. Similar results were observed with these analyses. The expression levels of beclin 1, ATG5, and LC3B were decreased, while the p62 level was increased in the A549/DDP-miR125b group compared with the A549/DDP-NC group (Figs. 7F–7H). Additionally, the miR-125 expression level was increased (Fig. 7I), while the BNIP3L and RORA levels were decreased in the A549/DDP-miR125b xenograft tumors (Figs. 7F–7H). Moreover, the *in vivo* protein expression levels of MDR-related genes p-gp and MVP (Figs. 7G and 7H) were downregulated by miR-125b overexpression. These results suggest that DDP in combination with miR-125b reverses resistance to DDP and significantly inhibits tumor growth *in vivo*, by regulating autophagy through suppressing the RORA/BNIP3L pathway.

## Discussion

Chemoresistance may be one of the contributors to the poor prognosis of LUAD. It has been well-accepted that miRNAs play crucial roles in cancer chemotherapeutic resistance. Therefore, miRNAs might have the potential to reverse resistance to drugs [15,16]. Fujita et al. [10] have found that the downregulation of miR-197 in platinum-resistant NSCLC specimens would lead to the promotion of chemoresistance, both *in vitro* and *in vivo*. Chen et al. [11] have demonstrated that a STAT3/PD-L1-dependent response would be seen for miR-526b-3p in reversing DDP resistance, suppressing metastasis, and activating CD8^+^ T lymphocytes. In this investigation, miR-125b was screened out as a DDP resistance-related gene by bioinformatics tools and experimental validation. Notably, the serum miR-125b levels were lower in LUAD patients with chemoresistance resistance, which was also correlated with a shorter survival period, indicating that miR-125 may be a potential biomarker for unsatisfied efficacy of platinum-based therapy and unfavorable prognosis in LUAD patients.

In recent years, the possible roles of miR-125b in multiple chemotherapy resistance have been documented. MiR-125b overexpression has been demonstrated to improve the sensitivity of MCF-7/R cells to doxorubicin in breast cancer [17] and significantly sensitized DDP-resistant colon cancer cells [18]. MiR-125b upregulation would promote cell death in the presence of DDP, and Bcl2 is the target of miR-125b, mediating its function in gallbladder cancer [19]. MiR-125b overexpression also increases the breast cancer cell sensitivity to tamoxifen [20]. This study indicated that miR-125b decreased cell proliferation and MDR-related protein levels. However, the miR-125b knocking down promoted cell proliferation and the MDR-related protein expression levels. Moreover, the miR-125b overexpression sensitized the A549/DDP cells to DDP, inhibiting tumor growth in xenograft mice. These findings indicate that miR-125b overexpression reverses DDP resistance, both *in vitro* and *in vivo*.

There is accumulating evidence that autophagy contributes to chemoresistance in cancer cells. Therefore, suppressing autophagy might be an effective therapeutic target to overcome chemoresistance. Peng et al. [21] have found that autophagy inhibitors, 3-MA, could restore the HeLa-R cells’ paclitaxel sensitivity in cervical cancer. This study found the miR-125b overexpression suppressed autophagy, whereas the miR-125b depletion activated autophagy in the A549/DDP cells. Moreover, miR-125b inhibited autophagy initiation but did not block autophagy flux. The results demonstrated that miR-125b inhibitor rescued the suppression of autophagy induced by 3-MA and BNIP3L siRNA, in turn promoting A549/DDP cell proliferation and resistance to DDP. Our results suggest that miR125b reverses DDP resistance by inhibiting autophagy in the A549/DDP cells.

However, the role of miR-125b in the autophagy process is still contradictory. Wang et al. [22] have found that miR-125b overexpression markedly increases autophagy through the Atg7 pathway, thereby sensitizing thyroid cancer cells to DDP treatment. A recent study has suggested that increasing autophagy may be the mechanism through which miR-125b confers resistance to 5-fluorouracil in colorectal cancer cells [23]. Xu et al. [24] have demonstrated that miR-125b induces autophagy by reducing the expression of Foxp3 through the TAK1/MKK4/cJNK/Smad axis in lung cancer. On the other hand, there are studies supporting our findings. Ren et al. [25] have shown that a mechanism involving miR-125b contributes to hepatocellular carcinoma cell resistance to chemotherapy by downregulating autophagy mediated by Eva-1 homolog A (EVA1A). MiR-125b overexpression has been shown to suppress colon cancer cell proliferation, invasion, and autophagy [26]. Wang et al. [27] have found that cell proliferation and autophagy suppression by MKNK2 knocking down would be almost eliminated by miR-125b inhibition in chemoresistant ovarian cancer cells. This contradiction may be due to the double-edged sword effects of autophagy for cancer therapy. On one hand, the tumor cells are shielded by autophagy from the negative effects of their surroundings. For example, autophagy would be activated when chemotherapeutic drugs invade. On the other hand, the tumor cells would be programmed to death by upregulating autophagy activity [28].

To further explore the potential mechanism of the effects of miR125b, online bioinformatics analysis tools were used to predict the possible target genes. Although no suitable candidate gene related to autophagy was found, several transcriptional factors were observed in the predicted target genes. Therefore, we turned to look for a transcriptional factor that regulated the autophagy-related gene BNIP3L, and finally, RORA was screened out. RORA is a critical clock gene that has been identified as a standalone predictive factor for overall survival in NSCLC [29]. Moreover, drug sensitivity and resistance have been linked to the core circadian clock genes [30]. Upon chemotherapy, ChIP-sequencing data revealed a loss of H3K27Ac at the RORA domain, the key transcription factor of epithelial phenotype [31]. However, reports about the role of RORA in cancer drug resistance have been rarely seen. After a series of experimental verification, our results manifested that miR-125b directly targeted RORA 3-UTR to inhibit the expressions of RORA and the downstream BNIP3L, and subsequently inhibited autophagy and reversed resistance in LUAD. Our current findings, however, require further investigation, to address the functions of other miRNAs involved in DDP resistance.

Taken together, our findings demonstrated that overexpressing miR-125b would reverse the DDP resistance of LUAD *in vitro* and *in vivo*, through modulating autophagy via targeting the RORA/BNIP3L axis. This study suggests that chemotherapy combined with miR-125b regulation might be a more effective treatment for DDP resistance.

## Supplementary Materials

**Table S1:** Information on the four chips from the gene expression omnibus database

Data source	Platform	Chip service provider	Sample size	Year
GSE56036	GPL15446	3D-Gene Human miRNA V17_1.0.0	Nine cisplatin-resistant and 9 cisplatin-sensitive tissue samples from 58 LUAD patients	2017
GSE168707	GPL29837	Qiagen miProfile Cancer miRNA qPCR Panel	Three cisplatin-resistant and 3 cisplatin-sensitive tissue samples of LUAD patients	2021
GSE43249	GPL14613	[miRNA-2] Affymetrix Multispecies miRNA-2 Array	Three pairs samples of A549 and A549/DDP	2017
GSE157692	GPL18573	Illumina NextSeq 500(*Homo sapiens*)	Two pairs samples of A549 and A549/DDP	2021

**Table S2:** Primer sequence used for RT-qPCR

Gene	Sequence
LC3B	F: TTCAGGTTCACAAAACCCGC
	R: TCTCACACAGCCCGTTTACC
P62	F: GACTACGACTTGTGTAGCGTC
	R: AGTGTCCGTGTTTCACCTTCC
beclin1	F: GGTGTCTCTCGCAGATTCATC
	R: TCAGTCTTCGGCTGAGGTTCT
BNIP3L	F: ATGTCGTCCCACCTAGTCGAG
	R: TGAGGATGGTACGTGTTCCAG
RORA	F: CACGACGACCTCAGTAACTACA
	R: TGGTGAACGAACAGTAGGGAA
GAPDH	F: GGAGCGAGATCCCTCCAAAAT
	R: GGCTGTTGTCATACTTCTCATGG
miR-125b	F: ACTGATAAATCCCTGAGACCCTAAC
	R: TATGGTTGTTCTGCTCTCTGTCAC
U6	F: CGCTTCGGCAGCCACATATAC
	R: TTCACGAATTTGCGTGTCATC

## Data Availability

Materials described in the manuscript, including all relevant raw data, will be freely available to any researcher wishing to use them for non-commercial purposes, without breaching participant confidentiality.
